# Metabolic engineering for improving anthranilate synthesis from glucose in *Escherichia coli*

**DOI:** 10.1186/1475-2859-8-19

**Published:** 2009-04-02

**Authors:** Víctor E Balderas-Hernández, Andrea Sabido-Ramos, Patricia Silva, Natividad Cabrera-Valladares, Georgina Hernández-Chávez, José L Báez-Viveros, Alfredo Martínez, Francisco Bolívar, Guillermo Gosset

**Affiliations:** 1 Departamento de Ingeniería Celular y Biocatálisis, Instituto de Biotecnología, Universidad Nacional Autónoma de México, Apdo Postal 510-3, Cuernavaca, Morelos, CP 62210, México; 2 Centro de Investigación en Biotecnología, Universidad Autónoma del Estado de Morelos, Av Universidad 2000, Cuernavaca, Morelos, CP 62210, México

## Abstract

**Background:**

Anthranilate is an aromatic amine used industrially as an intermediate for the synthesis of dyes, perfumes, pharmaceuticals and other classes of products. Chemical synthesis of anthranilate is an unsustainable process since it implies the use of nonrenewable benzene and the generation of toxic by-products. In *Escherichia coli *anthranilate is synthesized from chorismate by anthranilate synthase (TrpED) and then converted to phosphoribosyl anthranilate by anthranilate phosphoribosyl transferase to continue the tryptophan biosynthetic pathway. With the purpose of generating a microbial strain for anthranilate production from glucose, *E. coli *W3110 *trpD9923*, a mutant in the *trpD *gene that displays low anthranilate producing capacity, was characterized and modified using metabolic engineering strategies.

**Results:**

Sequencing of the *trpED *genes from *E. coli *W3110 *trpD9923 *revealed a nonsense mutation in the *trpD *gene, causing the loss of anthranilate phosphoribosyl transferase activity, but maintaining anthranilate synthase activity, thus causing anthranilate accumulation. The effects of expressing genes encoding a feedback inhibition resistant version of the enzyme 3-deoxy-D-*arabino*-heptulosonate-7-phosphate synthase (*aroG*^fbr^), transketolase (*tktA*), glucokinase (*glk*) and galactose permease (*galP*), as well as phosphoenolpyruvate:sugar phosphotransferase system (PTS) inactivation on anthranilate production capacity, were evaluated. In shake flask experiments with minimal medium, strains W3110 *trpD9923 *PTS^- ^and W3110 *trpD9923*/pJLB*aroG*^fbr^*tkt*A displayed the best production parameters, accumulating 0.70–0.75 g/L of anthranilate, with glucose-yields corresponding to 28–46% of the theoretical maximum. To study the effects of extending the growth phase on anthranilate production a fed-batch fermentation process was developed using complex medium, where strain W3110 *trpD9923/*pJLB*aroG*^fbr^*tkt*A produced 14 g/L of anthranilate in 34 hours.

**Conclusion:**

This work constitutes the first example of a microbial system for the environmentally-compatible synthesis of anthranilate generated by metabolic engineering. The results presented here, including the characterization of mutation in the *trpD *gene from strain W3110 *trpD9923 *and the development of a fermentation strategy, establish a step forward towards the future improvement of a sustainable process for anthranilate production. In addition, the present work provides very useful data regarding the positive and negative consequences of the evaluated metabolic engineering strategies.

## Background

**Figure 1:**
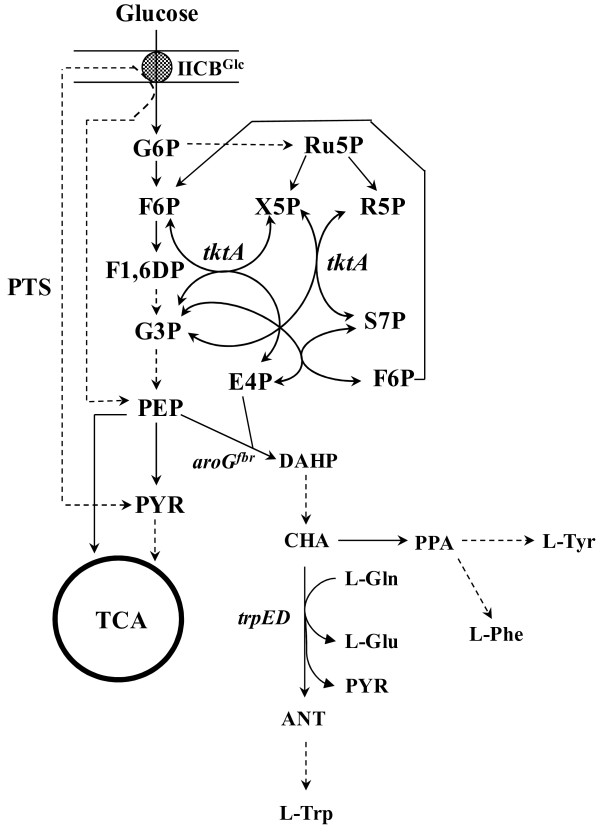
**Metabolic network related to anthranilate biosynthesis in *E. coli***. Arrows with dashed lines indicate more than one enzymatic reaction. Metabolite symbols: G6P, glucose 6-phosphate; F6P, fructose 6-phosphate; F1,6DP fructose 1,6 diphosphate; G3P, glyceraldehyde 3-phosphate; Ru5P, ribulose 5-phosphate; R5P, ribose 5-phosphate; X5P, xylulose 5-phosphate; S7P, sedoheptulose 7-phosphate; PYR, pyruvate; PEP, phosphoenolpyruvate; E4P, erythrose 4-phosphate; DAHP, 3-deoxy-D-*arabino*-heptulosonate 7-phosphate; CHA, chorismate; PPA, prephenate; ANT, anthranilate; L-Gln, L-glutamine; L-Glu, L-glutamate; L-Phe, L-phenylalanine; L-Tyr, L-tyrosine; L-Trp, L-tryptophan. Protein and gene symbols: IICB^Glc^, glucose-specific integral membrane permease; TCA, tricarboxylic acid cycle; PTS, phosphotransferase transport system; *tktA*, transketolase; *aroG*^fbr^, feedback inhibition resistant DAHP synthase; *trpED*, anthranilate synthase-phosphoribosyl transferase complex.

**Figure 2:**
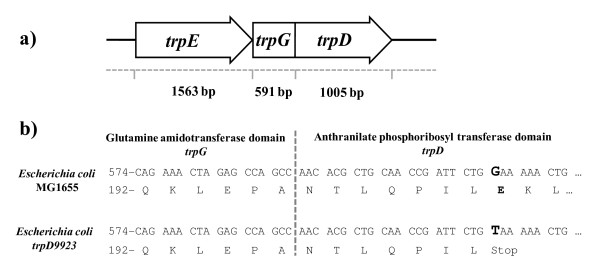
**Sequence determination of the *trpEGD *genes of *E. coli trpD9923***. (a) Organization of *trpEGD *genes of *E. coli*. (b) Comparison of the nucleotide and amino acid partial sequences of *trpGD *genes of *E. coli *MG1655 and *E. coli trpD9923*.

Anthranilate is an aromatic amine used as precursor for the synthesis of compounds having applications in the chemical, food and pharmaceutical industries. Current anthranilate manufacture methods are based on chemical synthesis using precursors derived from petroleum, such as benzene. Also, chemical synthesis of anthranilate is a multistep process requiring conditions of high temperature and pressure, which makes the process expensive for commercial use [1,2]. Several microbial and plant species have the metabolic capacity to synthesize this aromatic compound, opening the possibility for generating sustainable technologies for anthranilate manufacture. This compound is a metabolic intermediate and therefore it is normally not accumulated. Anthranilate is an intermediate in the tryptophan biosynthetic pathway (Fig. 1). Carbon flow into the common aromatic pathway starts with the condensation of D-erythrose 4-phosphate (E4P) and phosphoenolpyruvate (PEP) to yield 3-deoxy-D-*arabino*-heptulosonate 7-phosphate (DAHP), in a reaction catalyzed by the enzyme DAHP synthase. After six more reactions, chorismate is synthesized, leading to a branch point where biosynthetic pathways for L-tryptophan (L-Trp), L-tyrosine (L-Tyr) and L-phenylalanine (L-Phe) originate. In *Escherichia coli*, the first two reactions in the L-Trp biosynthetic pathway are catalyzed by the enzyme complex anthranilate synthase-phosphoribosyl transferase (TrpE-TrpD). It is a multifunctional and heterotetrameric complex composed of two TrpE and two TrpD polypeptides (component I and II, respectively). Component I (TrpE) catalyses the conversion of chorismate and glutamine to anthranilate, glutamate and pyruvate. The anthranilate synthase activity is the result of aminase and amidotransferase activities that are encoded by *trpE *and the amino terminal region encoded by *trpGD*, respectively (Fig. 2a). Component II (TrpD) catalyses the transfer of the phosphoribosyl group of 5-phosphorylribose-l-pyrophosphate to anthranilate, forming N-phosphoribosylanthranilate. The carboxyl terminal region of TrpD has the anthranilate phosphoribosyl transferase activity [3,4]. After five more metabolic steps, L-Trp is synthesized.

Early studies on the polarity of the L-Trp operon in *E. coli *enabled the identification of mutants that secreted anthranilate [5]. The characterization of one of the strains obtained by UV mutagenesis (W3110 *trpD9923*), revealed that the mutation was present in the *trpD *gene. These results suggest the feasibility of modifying *E. coli *to generate strains for anthranilate production. With the purpose of exploring a rational approach to improve the production capacity of strain W3110 *trpD9923*, in this work we characterized the mutation enabling anthranilate accumulation. In addition, we studied the effect of overexpressing genes encoding a feedback inhibition resistant DAHP synthase (*aroG*^fbr^), transketolase (*tktA*), glucokinase (*glk*) and galactose permease (*galP*) on anthranilate productivity and yield from glucose in strains having either active or inactive PEP:sugar phosphotransferase system (PTS).

## Results

### Characterization of *E. coli *W3110 *trpD9923*

The W3110 *trpD9923 *strain belongs to a set of *E. coli *mutants obtained after random mutagenesis by UV light exposure, having mutations in the first three genes of the tryptophan operon [5]. This report indicated that this strain is a tryptophan auxotroph which accumulates anthranilate. However, the specific mutation responsible for this phenotype and the anthranilate production capacity were not determined.

In order to characterize the mutation that causes anthranilate accumulation in this strain, the nucleotide sequence of the *trpEGD *genes was determined and compared to the corresponding sequence from *E. coli *MG1655 [6]. This analysis revealed a mutation at position 613, corresponding to the eighth codon of the anthranilate phosphoribosyl transferase domain of the anthranilate synthase component II (*trpD*), where a G to T transversion was detected, resulting in the generation of a stop codon (Fig. 2b). This mutation in *trpD9923 *results in the synthesis of a truncated anthranilate synthase component II protein, retaining the full glutamine amidotransferase domain and only seven of the 333 amino acid residues of the anthranilate phosphoribosyl transferase domain (Fig. 2b). This mutation in the *trpD *gene causes the loss of anthranilate phosphoribosyl transferase activity, but glutamine amidotransferase activity is not affected. Therefore, anthranilate can be synthesized in this strain, but it is not further metabolized to N-phosphoribosylanthranilate, thus causing anthranilate accumulation and tryptophan auxotrophy.

**Figure 3:**
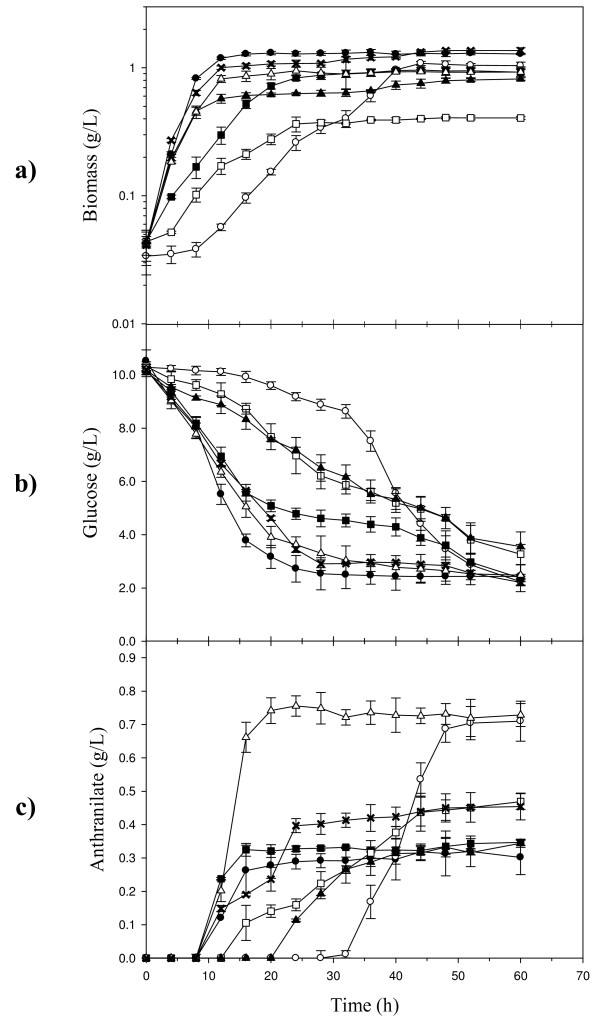
**Flask cultures of *E. coli *W3110 *trpD9923 *derivative strains for the production of anthranilate**. (a) Growth curves, (b) glucose consumption, and (c) anthranilate production. (filled circle) W3110 *trpD9923*; (open circle) W3110 *trpD9923 *PTS^-^; (filled square) W3110 *trpD9923 *PTS^-^/pv5Glk5GalP; (X) W3110 *trpD9923*/pJLB*aroG*^fbr^; (open triangle) W3110 *trpD9923*/pJLB*aroG*^fbr^*tkt*A; (open square) W3110 *trpD9923 *PTS^-^/pJLB*aroG*^fbr^*tkt*A; (filled triangle) W3110 *trpD9923 *PTS^-^/pv5Glk5GalP/pJLB*aroG*^fbr^*tkt*A. Graphs show results from the mean of the triplicate experiments.

To determine the anthranilate production capacity of strain W3110 *trpD9923*, cultures were performed in shake flasks with M9 mineral medium supplemented with 20 μg/mL tryptophan and 10 g/L of glucose at 37°C. Under these conditions, this strain displayed a specific growth rate (μ) of 0.26 ± 0.04 h^-1^(Table 1), a maximum biomass concentration of 1.29 ± 0.03 g_DCW_/L in 16 h and no lag phase was observed (Fig. 3a). The specific glucose consumption rate (*q*_Glc_) was 0.34 ± 0.01 g_Glc_/g_DCW_·h. After a 12 h production phase, this strain accumulated 0.31 ± 0.01 g/L of anthranilate as the maximum concentration (Fig. 3c) with a specific anthranilate production rate (*q*_Ant_) of 0.02 ± 0.00 g_Ant_/g_DCW_·h and an anthranilate yield from glucose (Y_Ant/Glc_) of 0.06 ± 0.01 g_Ant_/g_Glc _(Table 1).

**Table 1:** Comparison of kinetic and fermentation parameters of *E. coli *W3110*trpD9923 *derivative strains in flask cultures

**Strain**	**Final biomass** (g_DCW_/L)	**μ** (h^-1^)	***q*_Glc_** (g_Glc_/g_DCW_·h)	***q*_Ant_** (g_Ant_/g_DCW_·h)	**Y_Biom/Glc_** (g_DCW_/g_Glc_)	**Y_Ant/Glc_** (g_Ant_/g_Glc_)	**Final anthranilate titer **(g/L)
**W3110 *trpD9923***	1.29 ± 0.03 [16 h]^a^	0.26 ± 0.04 [0–12 h]^b^	0.34 ± 0.01 [0–16 h]	0.02 ± 0.00 [8–20 h]	0.18 ± 0.01 [0–16 h]	0.06 ± 0.01 [8–20 h]	0.31 ± 0.01
**W3110*trpD9923*PTS^-^**	1.04 ± 0.05 [44 h]	0.09 ± 0.01 [24–36 h]	0.13 ± 0.01 [0–44 h]	0.03 ± 0.00 [28–52 h]	0.18 ± 0.01 [0–44 h]	0.12 ± 0.01 [28–52 h]	0.70 ± 0.07
**W3110 *trpD9923*PTS^-^/pv5Glk5GalP**	0.94 ± 0.05 [32 h]	0.20 ± 0.01 [8–16 h]	0.24 ± 0.03 [0–20 h]	0.04 ± 0.00 [8–16 h]	0.15 ± 0.02 [0–20 h]	0.12 ± 0.00 [8–16 h]	0.33 ± 0.01
**W3110 *trpD9923*/pJLB*aroG*^fbr^**	1.09 ± 0.01 [16 h]	0.20 ± 0.00 [0–12 h]	0.30 ± 0.01 [0–12 h]	0.07 ± 0.00 [4–12 h]	0.28 ± 0.01 [0–12 h]	0.16 ± 0.01 [4–12 h]	0.44 ± 0.00
**W3110 *trpD9923*/pJLB*aroG*^fbr^*tkt*A**	0.93 ± 0.04 [20 h]	0.24 ± 0.00 [0–12 h]	0.37 ± 0.02 [0–16 h]	0.07 ± 0.00 [8–20 h]	0.17 ± 0.01 [0–16 h]	0.20 ± 0.05 [8–20 h]	0.75 ± 0.04
**W3110 *trpD9923*PTS^-^/pJLB*aroG*^fbr^*tkt*A**	0.39 ± 0.00 [32 h]	0.15 ± 0.01 [4–16 h]	0.43 ± 0.04 [0–24 h]	0.03 ± 0.00 [12–44 h]	0.10 ± 0.01 [0–24 h]	0.10 ± 0.02 [12–44 h]	0.45 ± 0.02
**W3110 *trpD9923*PTS^-^/pv5Glk5GalP/pJLB*aroG*^fbr^*tkt*A**	0.79 ± 0.03 [24 h]	0.24 ± 0.00 [4–12 h]	0.21 ± 0.00 [0–28 h]	0.020 ± 0.00 [20–40 h]	0.19 ± 0.00 [0–28 h]	0.14 ± 0.03 [20–40 h]	0.33 ± 0.02

^a ^values in brackets indicate the time maximum biomass was achieved ^b ^values in brackets indicate the period considered for calculation of kinetic parameters.

### PTS inactivation in strain *trpD9923*

To improve the anthranilate production capacity of W3110 *trpD9923*, two different and complementary metabolic engineering strategies were applied: one involved increasing the availability of PEP and E4P; two metabolic precursors for anthranilate biosynthesis, and the other was based on redirecting carbon flow from central metabolism into the common aromatic pathway. Condensation of PEP and E4P is the first step in the aromatic amino acid biosynthesis pathway (Fig. 1). Several reported studies have demonstrated that PEP is a limiting precursor with regard to aromatics yield from glucose [7-9]. When *E. coli *is growing with glucose as the carbon source, PTS is the main activity that consumes PEP; therefore, it has been identified as a target for inactivation to increase aromatics production capacity [10-12]. In order to increase PEP biosynthetic availability in the cell, the PTS operon was inactivated in strain W3110 *trpD9923 *by transduction of the Δ*ptsHIcrr*::km^R ^mutation, generating strain W3110 *trpD9923 *PTS^-^. Flask cultures with this PTS^- ^strain using M9 mineral medium showed a significantly different growth profile compared to that observed with the PTS^+ ^strain W3110 *trpD9923*. Cultures with strain W3110 *trpD9923 *PTS^- ^showed a 10 h lag phase and the maximum biomass was 1.04 ± 0.05 g_DCW_/L in 44 h (Fig. 3a). This diminished growth capacity was evident by a 65% lower μ than that observed for W3110 *trpD9923 *(Table 1). Also, glucose consumption rate in the PTS^- ^strain was 62% lower than the *q*_Glc _of the progenitor strain. W3110 *trpD9923 *PTS^- ^displayed a 24 h anthranilate production phase, where the *q*_Ant _was 1.5-fold higher and the Y_Ant/Glc _2-fold higher than the corresponding values obtained in the PTS^+ ^strain cultures (Table 1). As a result, the PTS^- ^strain accumulated a 2.2-fold higher amount of anthranilate than W3110 *trpD9923 *(Fig. 3c). These results show that PTS inactivation caused a positive effect on anthranilate production capacity.

### Increasing glucose transport capacity in strain W3110 *trpD9923 *PTS^-^

As expected, inactivation of PTS in W3110 *trpD9923 *caused a significant decrease in its *q*_Glc_, due to a reduced capacity to import this sugar [12]. As a result, its growth rate was severely affected. Thus, in order to increase the glucose transport capacity, strain W3110 *trpD9923 *PTS^- ^was transformed with plasmid pv5Glk5GalP, which carries the genes *glk *and *galP *encoding glucokinase (Glk) and galactose permease (GalP), respectively. Expression of these two proteins has been shown to restore glucose import and phosphorylation activities; functions previously provided by the PTS [13]. Shake flask experiments with strain W3110 *trpD9923 *PTS^-^/pv5Glk5GalP under previously described conditions showed that expression of *glk *and *galP *in the PTS^- ^strain caused a positive effect in glucose assimilation capacity; the observed *q*_Glc _was 1.8-fold higher than the value for the PTS^- ^strain (Table 1). Also, the μ increased 2.2-fold with respect to that observed for the PTS^- ^strain. Values for Y_Ant/Glc _were similar to those in the PTS^- ^strain and *q*_Ant _increased 1.3-fold (Table 1). However, the anthranilate production phase was reduced to 8 h due to faster glucose consumption; therefore, a lower anthranilate titer of 0.33 ± 0.01 g/L was reached at the end of the culture (Fig. 3c).

### Redirection of glycolytic and pentose phosphate pathway precursors to the common aromatic amino acid biosynthetic pathway

As mentioned before, condensation of PEP and E4P generates DAHP by action of DAHP synthase (Fig. 1). However this enzyme is highly regulated by allosteric control. Thus, to increase cellular DAHP synthase activity, strain W3110 *trpD9923 *was transformed with plasmid pJLB*aroG*^fbr ^[14], which harbors the *aroG*^fbr ^gene encoding a feedback inhibition resistant mutant of DAHP synthase. Increased dosage of *aroG*^fbr ^caused an increase in the *q*_Ant _and Y_Ant/Glc _(3.5 and 2.7-fold, respectively), and 1.4-fold higher anthranilate accumulation in comparison with the parental strain. Increasing DAHP synthase activity causes a higher demand for E4P; therefore, to avoid a limitation for this intermediate, it is necessary to increase the activity of the enzyme that synthesizes it. A way to achieve this is through the high level expression of the enzyme transketolase, responsible for E4P production. Therefore, to evaluate the effect of the co-expression of *tktA *on anthranilate production, this gene was cloned downstream of the *aroG*^fbr ^gene, generating the plasmid pJLB*aroG*^fbr^*tktA*. Co-expression of *aroG*^fbr ^and *tktA *in strain W3110 *trpD9923 *did not affect significantly the μ, *q*_Glc_, and *q*_Ant _parameters, in comparison with strain W3110 *trpD9923/*pJLB*aroG*^fbr^. However, the presence of *tktA *gene in the plasmid pJLB*aroG*^fbr ^caused a 1.2-fold increase in Y_Ant/Glc_, resulting in a 1.7-fold higher anthranilate final titer (Table 1) with respect to the strain expressing only *aroG*^fbr^. Although the final anthranilate titer accumulated by W3110 *trpD9923*/pJLB*aroG*^fbr^*tktA *is comparable to that produced by W3110 *trpD9923 *PTS^-^, the *q*_Ant _of the former strain is 2.3-fold higher (Table 1). The maximum theoretical yield (^max^Y_Ant/Glc_) of anthranilate from glucose is 0.435 g_Ant_/g_Glc_, considering this value, the Y_Ant/Glc _from W3110 *trpD9923*/pJLB*aroG*^fbr^*tktA *strain corresponded to 46% of the ^max^Y_Ant/Glc_.

Previous results demonstrated that the simultaneous expression of *aroG*^fbr ^and *tktA *genes caused a 3.3-fold increase in Y_Ant/Glc _and a 2.4-fold increase in the anthranilate titer in strain W3110 *trpD9923*, thus, in order to increase carbon flux into aromatic biosynthesis and E4P availability, strains W3110 *trpD9923 *PTS^- ^and W3110 *trpD9923 *PTS^-^/pv5Glk5GalP were transformed with plasmid pJLB*aroG*^fbr^*tktA*. The presence of plasmid pJLB*aroG*^fbr^*tktA *in strain W3110 *trpD9923 *PTS^- ^had a negative impact on the final biomass concentration corresponding to 37% of the strain lacking this plasmid. When compared to W3110 *trpD9923 *PTS^-^, no significant changes in *q*_Ant _and Y_Ant/Glc _were detected. However the final anthranilate titer was 0.45 ± 0.02 g/L due to the lower biomass concentration (Fig. 3c). Transformation of strain W3110 *trpD9923 *PTS^-^/pv5Glk5GalP with plasmid pJLB*aroG*^fbr^*tktA *did not have a significant effect on its growth capacity and the *q*_Glc_. In contrast, the *q*_Ant _decreased 2-fold but the production phase was 2.5-fold longer than that from the isogenic strain lacking pJLB*aroG*^fbr^*tktA*, therefore, similar final anthranilate titers were produced by both strains (Table 1).

### Fed-batch fermentor cultures for anthranilate production

**Figure 4:**
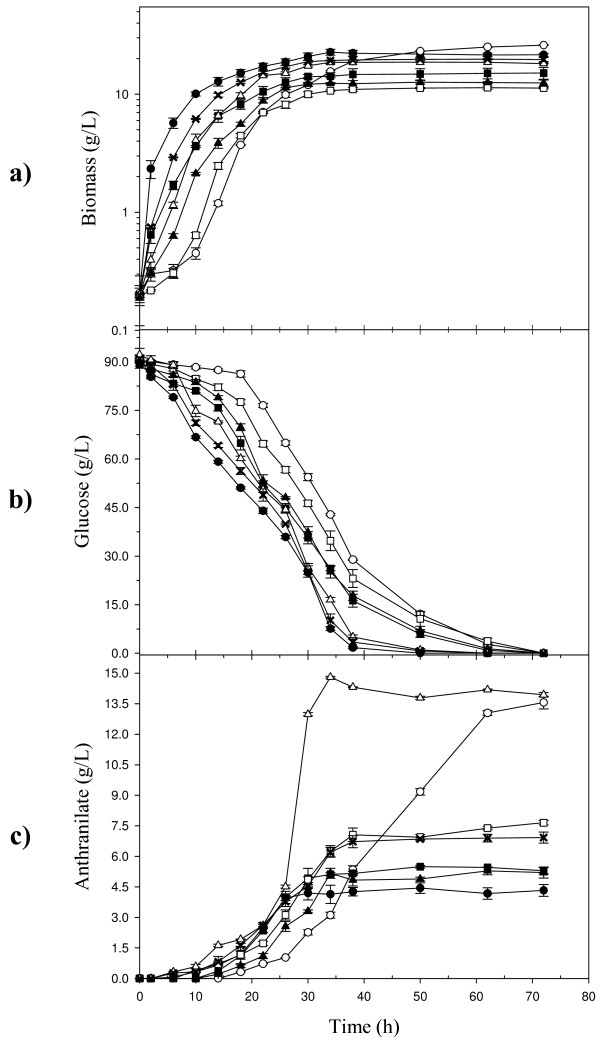
**Fermentor cultures of *E. coli *W3110 *trpD9923 *derivative strains for the production of anthranilate**. (a) Growth curves, (b) glucose consumption, and (c) anthranilate production. (filled circle) W3110 *trpD9923*; (open circle) W3110 *trpD9923 *PTS^-^; (filled square) W3110 *trpD9923 *PTS^-^/pv5Glk5GalP; (X) W3110 *trpD9923*/pJLB*aroG*^fbr^; (open triangle) W3110 *trpD9923*/pJLB*aroG*^fbr^*tkt*A; (open square) W3110 *trpD9923 *PTS^-^/pJLB*aroG*^fbr^*tkt*A; (filled triangle) W3110 *trpD9923 *PTS^-^/pv5Glk5GalP/pJLB*aroG*^fbr^*tkt*A. Graphs show results from the mean of the duplicate experiments.

Previous results indicated that anthranilate accumulation occurs mainly during the growth phase in all studied strains. Therefore, to study the effect of extending the growth phase on anthranilate production, all strains were cultured in a fermentor using a fed-batch system with complex medium where a total of 30 g/L yeast extract and 90 g/L glucose were fed in order to improve the final biomass concentration. As Figure 4 shows, all strains displayed growth, glucose consumption and anthranilate accumulation profiles similar to those observed in the flask cultures (Fig. 3). By using a fed-batch process, final biomass concentration was increased an average of 19-fold among all strains (Fig. 4a), when compared to shake-flask conditions (Fig. 3a), likewise, the anthranilate production phase and final anthranilate titer were increased an average of 1.6-fold and 19.4-fold (Fig. 4c), respectively. Analysis of kinetic parameters (Table 2) of all fermentor cultures demonstrated that W3110 *trpD9923*/pJLB*aroG*^fbr^*tktA *was the best anthranilate producer strain. It accumulated 14 g/L of anthranilate in 34 h with a Y_Ant/Glc _of 0.20 ± 0.00 g_Ant_/g_Glc_, the highest values observed among all W3110 *trpD9923 *derivatives (Table 2). It should be noted that the Y_Ant/Glc _values presented in Table 2 are useful only for comparison among strains grown in the fed batch conditions, since nutrients present in the yeast extract could provide precursors for anthranilate synthesis. With respect to acetic acid production, final titer in W3110 *trpD9923 *strain was 9.65 ± 2.17 g/L (Table 2). In contrast, a much lower amount of acetic acid (0.50 ± 0.1 g/L) was detected in the medium of W3110 *trpD9923*/pJLB*aroG*^fbr^*tktA *cultures. In addition, PTS inactivation caused a severe reduction in the production of acetic acid, as it was not detected in the supernatants of all PTS^- ^strains (Table 2).

**Table 2:** Comparison of kinetic and fermentation parameters of *E. coli *W3110*trpD9923 *derivative strains in fed-batch fermentor cultures

**Strain**	**Final biomass** (g_DCW_/L)	**μ** (h^-1^)	***q*_Glc_** (g_Glc_/g_DCW_·h)	***q*_Ant_** (g_Ant_/g_DCW_·h)	**Y_Biom/Glc_** (g_DCW_/g_Glc_)	**Y_Ant/Glc_** (g_Ant_/g_Glc_)	**Final anthranilate titer** (g/L)	**Final acetate titer** (g/L)
**W3110 *trpD9923***	21.35 ± 3.76 [34 h]^a^	0.25 ± 0.02 [2–10 h]^b^	0.17 ± 0.01 [0–34 h]	0.01 ± 0.00 [6–30 h]	0.27 ± 0.02 [0–34 h]	0.08 ± 0.01 [6–30 h]	4.21 ± 0.03	9.65 ± 2.17
**W3110 *trpD9923*PTS^-^**	23.30 ± 0.56 [62 h]	0.10 ± 0.00 [6–18 h]	0.06 ± 0.00 [0–62 h]	0.01 ± 0.00 [14–72 h]	0.28 ± 0.00 [0–62 h]	0.15 ± 0.00 [14–72 h]	13.30 ± 0.89	ND^c^
**W3110 *trpD9923*PTS^-^/pv5Glk5GalP**	14.66 ± 1.26 [38 h]	0.15 ± 0.00 [6–18 h]	0.13 ± 0.02 [0–38 h]	0.01 ± 0.00 [10–34 h]	0.20 ± 0.03 [0–38 h]	0.09 ± 0.00 [10–34 h]	5.30 ± 0.07	ND
**W3110 *trpD9923*/pJLB*aroG*^fbr^**	19.47 ± 0.12 [34 h]	0.21 ± 0.00 [2–14 h]	0.12 ± 0.01 [0–30 h]	0.01 ± 0.00 [2–38 h]	0.28 ± 0.02 [0–30 h]	0.08 ± 0.002 [2–38 h]	6.72 ± 0.01	3.18 ± 0.52
**W3110 *trpD9923*/pJLB*aroG*^fbr^*tkt*A**	18.54 ± 0.19 [34 h]	0.18 ± 0.01 [6–18 h]	0.14 ± 0.01 [0–34 h]	0.02 ± 0.00 [2–34 h]	0.24 ± 0.02 [0–34 h]	0.20 ± 0.00 [2–34 h]	14.00 ± 0.07	0.50 ± 0.1
**W3110 *trpD9923*PTS^-^/pJLB*aroG*^fbr^*tkt*A**	11.12 ± 0.25 [38 h]	0.13 ± 0.00 [10–26 h]	0.16 ± 0.01 [0–34 h]	0.02 ± 0.00 [6–38 h]	0.19 ± 0.02 [0–34 h]	0.11 ± 0.00 [6–38 h]	7.16 ± 0.53	ND
**W3110 *trpD9923*PTS^-^/pv5Glk5GalP/pJLB*aroG*^fbr^*tkt*A**	15.40 ± 0.14 [38 h]	0.16 ± 0.00 [10–22 h]	0.15 ± 0.01 [0–30 h]	0.02 ± 0.01 [10–34 h]	0.23 ± 0.02 [0–30 h]	0.09 ± 0.01 [10–34 h]	5.08 ± 0.06	ND

^a ^values in brackets indicate the time maximum biomass was achieved ^b ^values in brackets indicate the period considered for calculation of kinetic parameters. ^c ^ND, non detectable levels.

## Discussion

In this work, molecular characterization of the *trpD9923 *mutant allele demonstrated that UV-light treatment generated a nonsense mutation in the *trpD *gene. As a result of this mutation, gene *trpD9923 *encodes a truncated anthranilate synthase component II, strongly suggesting that the mutant protein retained glutamine amidotransferase activity and lost the anthranilate phosphoribosyl transferase function. This assumption is consistent with the observed phenotype of strain W3110 *trpD9923 *(anthranilate accumulation and L-Trp auxotrophy). The identification of the locus and the type of mutation present in strain W3110 *trpD9923 *will facilitate future efforts for the construction of anthranilate production strains by enabling the generation or transfer of this mutant allele to different microbial species.

Cultures in shake flask and fermentor allowed the characterization of strain W3110 *trpD9923 *and derivatives with genetic modifications expected to have an impact on anthranilate production capacity. Under the fed-batch conditions utilized in this work, strain W3110 *trpD9923 *produced 4.2 g/L of anthranilate. With the purpose of improving its performance as a production strain, W3110 *trpD9923 *was subjected to genetic modifications, following several metabolic engineering strategies expected to improve microbial strains for the production of aromatic amino acids, and more recently in the production of chorismate-derived fine chemicals [15-17]. A key target for improving aromatic amino acids production capacity is the modification of central metabolism to increase PEP and E4P availability [7-9]. Fifty percent of the PEP generated in glycolysis is spent in glucose uptake by the PTS. As the major PEP consuming activity in *E. coli*, PTS is the main target for inactivation to increase precursor availability for aromatic compounds [10,11]. PTS inactivation in W3110 *trpD9923 *caused a 3.2-fold increase in the anthranilate titer in fed batch cultures. Also, PTS inactivation caused a severe reduction in acetic acid production in comparison with the PTS^+ ^strain, eliminating the negative effect of acetate accumulation that is responsible for growth and productivity reduction [18-20].

However, an expected consequence of PTS inactivation was a reduction in *q*_Glc_, resulting in 60% lower growth rate of W3110 *trpD9923 *PTS^-^. It has been demonstrated that expression of *galP *and *glk *genes increases glucose internalization and glycolytic flux to fermentation products in PTS^- ^mutants [10,13,21,22]. In W3110 *trpD9923 *PTS^- ^the presence of plasmid pv5Glk5GalP effectively increased glucose assimilation capacity as was evident by the higher values of μ and *q*_Glc _than those present in W3110 *trpD9923 *PTS^-^strain. However, in fed batch cultures, Y_Ant/Glc _and anthranilate titer were reduced in W3110 *trpD9923 *PTS^-^/pv5Glk5GalP. A similar effect was reported by Chen et al. [23], where *galP *expression was ineffective in increasing the L-Phe titers in an *E. coli *PTS^- ^strain. A possible explanation for this result is that glucose imported by GalP must be phosphorylated by glucokinase, using ATP as the phosphate donor, thus possibly having a negative impact on the cell's energy balance, growth capacity and productivity. An alternate explanation is that the lower *q*_Glc _alters carbon flux distribution, resulting in a negative impact on biosynthetic metabolism. This negative effect was evident by the affected growth capacity of PTS^- ^strain and also by the lower values of Y_Biom/Glc _observed in strains PTS^- ^transformed with plasmid pv5Glk5GalP and/or pJLB*aroG*^fbr^*tkt*A (Tables 1 and 2). Metabolic flux redirection in several segments of central metabolism has been reported as a consequence of PTS inactivation and the corresponding lower glucose transport capacity in *E. coli *[24].

As mentioned, inactivation of PTS is a common modification to improve aromatics biosynthesis in *E. coli*. The PTS^- ^strains are always further modified to increase their glucose transport capacity, either by isolating spontaneous mutants of by expressing genes encoding alternate glucose transport and phosphorylating activities [10,22]. In this work, it was found that the residual glucose transport capacity of a PTS^- ^strain is sufficient to allow relatively high anthranilate production capacity. In both shake flask and fermentor cultures, strain W3110 *trpD9923 *PTS^- ^displayed the second highest final anthranilate titer of all studied strains; however, the productivity was low. When the *q*_Glc _and growth capacity were improved in this strain by the expression of *galP *and *glk *genes, the final anthranilate titer was reduced. These results suggest that fine-tuning the expression level of *galP *and *glk *could allow the development of PTS^- ^production strains having both adequate growth and anthranilate production capacities.

In a wild type *E. coli *strain, carbon flow into the common aromatic pathway represents only 1.5% of the glucose uptake rate [24]. This is the result of tight regulation of the DAHP synthase isozymes that control carbon entry into this pathway [15]. To overcome this limitation, feedback resistant mutant versions of either one of the three DAHP synthase isozymes have been expressed in engineered aromatics production strains [8,14,25,26]. In the present work, expression of the feedback resistant DAHP synthase *aroG*^fbr ^in fermentor cultures resulted in a 1.6-fold higher amount of anthranilate accumulated in comparison to W3110 *trpD9923 *strain, also higher values of *q*_Ant _and Y_Ant/Glc _were achieved. In addition to *aroG*^fbr ^expression, overexpression of the non-oxidative pentose pathway enzyme; transketolase, has been shown to increase E4P availability [27]. The co-expression of *aroG*^fbr ^and *tktA *genes in strain W3110 *trpD9923 *resulted in elevated titers of anthranilate; 14 g/L were obtained under fed-batch fermentor culture. Also, the highest *q*_Ant _and Y_Ant/Glc _values of strain W3110 *trpD9923*/pJLB*aroG*^fbr^*tkt*A evidenced the elevated carbon flux redirection from central metabolism to the product-forming pathway *via *DAHP. Analysis of kinetic and fermentation parameters from flask cultures of W3110 *trpD9923*/pJLB*aroG*^fbr ^and W3110 *trpD9923*/pJLB*aroG*^fbr^*tkt*A, when compared to W3110 *trpD9923*, enabled to determine that overexpression of *aroG*^fbr ^and *tktA *contributes with 80% and 20% of the increase in Y_Ant/Glc_, respectively. Co-expression of *tktA *in other *E. coli *engineered strains has shown a 30–40% increment in aromatic products yields [25,28-30]. It was also observed that co-expression of *aroG*^fbr ^and *tkt*A had an effect in acetic acid production. The expression of *aroG*^fbr ^caused a 3-fold reduction in final acetic acid titer when compared to W3110 *trpD9923*. Remarkably, the presence of plasmid pJLB*aroG*^fbr^*tkt*A in strain W3110 *trpD9923 *caused a 19.3-fold reduction in the final acetate concentration. This result can be explained considering that redirection of PEP to the common aromatic pathway should reduce carbon flow to pyruvate, an intermediate that is both a direct and indirect precursor to acetic acid.

The presence of plasmid pJLB*aroG*^fbr^*tkt*A in all W3110 *trpD9923 *derivatives caused an increase in *q*_Ant_. However, the simultaneous presence of compatible plasmids pJLB*aroG*^fbr^*tkt*A and pv5Glk5GalP in W3110 *trpD9923 *PTS^- ^resulted in low final biomass concentration, possibly caused by plasmid and gene expression metabolic burden. This negative effect was more pronounced in the *trpD9923 *PTS^-^/pJLB*aroG*^fbr^*tkt*A strain, possibly due to a carbon and energy limited condition caused by its lower glucose import capacity resulting from an inactive PTS. Co-expression of *aroG*^fbr ^and *tkt*A genes caused a reduction in the Y_Biom/Glc _of 11% in the PTS^+ ^strain and 32% in the PTS^-^strain in comparison with their parental strains without pJLB*aroG*^fbr^*tkt*A plasmid (Tables 1 and 2). These results suggest that the lower anthranilate titers observed in the *trpD9923 *PTS^-^/pJLB*aroG*^fbr^*tkt*A strain is consequence of the susceptibility of the PTS^- ^strain to the metabolic burden caused by gene overexpression.

The microbial synthesis of anthranilate has been previously described using a *Bacillus subtillis *strain resistant to sulfaguanidine and flourotryptophan [31]. It is reported that fermentor cultures with this strain using minimal medium, resulted in the production on 3.5 g/L of anthranilate and 25 g/L of acetoin after 60 h. In contrast, fermentor cultures using complex medium with strain W3110 *trpD9923*/pJLB*aroG*^fbr^*tkt*A produced 14 g/L of anthranilate in 34 h and a low level of acetate was detected (0.50 g/L).

The results presented in this work, including the characterization of mutation *trpD9923 *and the effects on strain productivity of specific genetic modifications, will enable further optimization work focused in exploring additional metabolic engineering strategies and process technology to improve the current *E. coli*-based production system for the environmentally-compatible synthesis of anthranilate. These efforts should include the evaluation of using environmentally-friendly raw materials such as lignocellulosic hydrolysates and other carbon sources; as glycerol, for the production of anthranilate in a sustainable process.

## Methods

### Strains and plasmids

Bacterial strains and plasmids used in this study are described in Table 3. *E. coli *strain W3110*trpD9923 *was obtained from the *E. coli *Genetic Stock Center (Yale University, New Haven, CT). *E. coli *W3110 *trpD9923 *strain is a mutant in the tryptophan operon obtained by treatment with ultraviolet radiation [3]; it is a tryptophan auxotroph. A PTS^- ^derivative of *trpD9923 *was obtained by P1 *vir *phage transduction using PB11 (Δ*ptsH*, *ptsI*, *crr*::Km^R^) strain as donor, as described by Flores et al [10].

**Table 3:** *Escherichia coli *strains and plasmids used in this work

**Strain/Plasmid**	**Characteristics**	**Reference**
**Strains**		
PB11	JM101 [*supE*, *thi*, Δ(*lac-proAB*), F'] Δ*ptsHI*, *crr*::km^R^, glucose^-^	[10]
W3110 *trpD9923*	W3110 [F^-^λ^- ^INV (*rrn*D-*rrn*E) 1] tryptophan auxotroph, randomly mutagenized by treatment with ultraviolet radiation.	[5]
W3110 *trpD9923 *PTS^-^	As W3110 *trpD9923 *but Δ*ptsHI*, *crr*::km^R^, glucose^-^	This work
		
**Plasmids**		
pJLB*aroG*^fbr^	*aroG*^fbr ^expressed from the *lacUV5 *promoter, *lacI*^q ^and *tet *genes, tetracycline resistance, pACYC184 replication origin.	[14]
pv5Glk5GalP	*glk *and *galP *genes expressed from the *trc5 *promoter, spectinomycin resistance. pCL1920 replication origin.	[13]
pJLB*aroG*^fbr^*tkt*A	pJLB*aroG*^fbr ^derivative, containing the *tktA *gene with its native promoter.	This work

Plasmid pJLB*aroG*^fbr ^carries the *aroG*^fbr ^gene encoding a feedback inhibition resistant mutant version of the enzyme DAHP synthase under transcriptional control of the *lac*UV5 promoter [14]. To co-express from this plasmid the gene encoding transketolase, the *tktA *gene including its native promoter region was amplified by PCR using chromosomal DNA of *E. coli *W3110 as template and the forward primer 5' GCGCAGCGGACGGGCGAGTAGAT**TGCGCA**3' and the reverse primer 5' CGCCTGTTCGTTATCTATTCCGC**ACGCGT**CGCG 3', both primers contain the *Fsp*I site (in bold). The *tktA *PCR product was cloned into plasmid pJLB*aroG*^fbr ^previously digested with *Bst*Z17I enzyme, to generate plasmid pJLB*aroG*^fbr^*tktA*. Plasmid pv5Glk5GalP carries the *glk *and *galP *genes, encoding glucokinase (Glk) and galactose permease (GalP), under transcriptional control of a *trc*-derived promoter [13].

### Nucleotide sequence determination of *trpED *genes

Chromosomal DNA (200 ng) from strain W3110*trpD9923 *was used as template for PCR amplification using a set of primers designed with the Clone Manager v6.0 software (Scientific and Educational Software, Durham, NC). The primers were designed to bind to different regions of the *trpED *genes, allowing the determination of the full sequence. Primers used were the following: 5'TAGAGAATAACCATGGAAACACAAAAACCG3', 5'CGCGGATCCCGGTTTGCATCATTTACCCTCG3', 5'CGATTACCAGCAGGCCTCCGGTTGCAGCGTGGTGGCTGGCTCTAG3', 5'ATTCCAGTTCCATCCGGAATCC3', 5'ATCTCGTTCGGGTGCTCACC3', 5'CAGGAGAAAGCATCAGCACC3' and 5'GAGTTCGGTGGCGTAGTGCG3'. PCR reactions were carried out with the Elongase enzyme mix (Invitrogen, Carlsbad, CA) in accordance with the supplier recommendations. PCR products were analyzed for expected size and purified using a PCR purification kit (Marligen, BioScience, Ijamsville, MD). Nucleotide sequences were determined from PCR templates by the Taq FS Dye Terminator Cycle Fluorescence-Based Sequencing method, with an Applied Biosystems Model 377-18 sequencer (Foster City, CA).

### Growth media, inoculum preparation and culture conditions

Cells were routinely grown in Luria Bertani (LB) broth or LB agar plates [32]. M9 mineral medium was used for flask cultures, containing 10 g/L glucose, 6 g/L Na_2_HPO_4_, 0.5 g/L NaCl, 3 g/L KH_2_PO_4_, 1 g/L NH_4_Cl, 246.5 mg/L MgSO_4_, 14.7 mg/L CaCl_2 _and 10 μg/mL vitamin B1, and supplemented with 20 μg/mL tryptophan. Medium for fermentor cultures contained 3 g/L Na_2_HPO_4_, 3 g/L KH_2_PO_4_, 1.7 g/L (NH_4_)_2_HPO_4 _and 1 mL/L of trace elements solution. This solution contains 27 g/L FeCl_3_, 2 g/L ZnCl_3_, CoCl_2_·6H_2_O, 2 g/L Na_2_MoO_4_·2H_2_O, 2 g/L CaCl_2_·2H_2_O, 0.5 g/L H_3_BO_3 _and 100 mL/L HCl.

Fermentor medium initially contained 10 g/L of yeast extract and 30 g/L of glucose. A total of two independent pulses containing 30 g/L glucose and 10 g/L yeast extract were added to the fermentor whenever glucose concentration in the medium decreased to 10 g/L. Each pulse contained 25 mL of 60% glucose solution and 25 mL of 20% yeast extract solution. Antibiotics were added to the corresponding cultures at a final concentration of 30 μg/mL spectomycin, 20 μg/mL tetracycline and 30 μg/mL kanamycin during selection, propagation and fermentation stages.

Inoculum preparation was started using strain samples from frozen vials that were cultured overnight at 37°C in M9 mineral medium plates supplemented with 0.2% of glucose and 20 μg/mL tryptophan, colonies from these plates were used to inoculate baffled shake flasks. For fermentor cultures, colonies from plates were grown in shake flasks with 50 mL LB medium, after overnight culture at 37°C a sample was used for inoculation.

Flask cultures were done in 250 mL flasks containing 50 mL of M9, inoculated at an initial optical density at 600 nm (OD_600 nm_) of 0.1 and incubated for 60 h at 37°C and 300 rpm in an orbital shaker (Series 25, New Brunswick Scientific, Inc., NJ).

Fermentor cultures were performed in 1 L stirred tank bioreactors (Applikon, The Netherlands), using a working volume of 500 mL. Cultures were inoculated at an initial OD_600 nm _of 0.5. pH was maintained at 7.0 by automatic addition of a 12.5% NH_4_OH solution. Temperature was controlled at 37°C. Airflow was set to 1 vvm. Dissolved oxygen tension was measured with a polarographic oxygen electrode (Applisens, Applikon) and maintained above 20% air saturation during all cultivation period by modifying the impeller speed.

For cultures of strains carrying plasmid pJLB*aroG*^fbr^, pJLB*aroG*^fbr^*tktA *and/or pv5Glk5GalP gene induction was started by adding IPTG to a final concentration of 0.1 mM at an OD_600 nm _of 0.6 for shake flask and 3.0 for fermentor cultures.

### Kinetic parameters calculation

For the characterization of the strains used in this work, specific rates of growth (μ), glucose consumption (*q*_Glc_), anthranilate production (*q*_Ant_), yield of biomass on glucose (Y_Biom/Glc_) and yield of anthranilate on glucose (Y_Ant/Glc_) were determined. μ, *q*_Glc _and Y_Biom/Glc _were calculated during exponential growth phase. Since growth rates and anthranilate production kinetics differed among studied strains, *q*_Ant _and Y_Ant/Glc _were calculated considering only the anthranilate production phase, defined as the time period starting one sample (1 hour) before anthranilate is detected up to the point when a sharp decrease in anthranilate accumulation was observed. Flask cultures were performed in triplicate and fermentor cultures in duplicate. The values reported represent the mean of the experiments performed.

### Analytical methods

Biomass concentration was measured as OD_600 nm _using a spectrophotometer (Beckman DU-70, Palo Alto, CA) and converted to dry cell weight (DCW) considering that 1 OD_600 nm _= 0.37 g_DCW_/L [13]. Samples taken during cultivation period were centrifugated at 10000 rpm for 2 min. Supernatant was filtered using 0.45 μm syringe-filter and stored at -20°C for subsequent analysis. Glucose was determined using an enzymatic analyzer (YSI 2700, YSI Life Sciences, OH). Acetate was determined by high performance liquid chromatography (HPLC) (Waters, Milford, MA), using an Aminex HPX-87H column (300 × 7.8 mm; Bio-Rad, Hercules, CA); running conditions were 5 mM H_2_SO_4 _as mobile phase, flow of 0.5 mL/min and temperature of 50°C. Detection was performed by photodiode array at 210 nm. Anthranilate was determined by HPLC (Agilent Technologies, Palo Alto, CA) using a Synergy Hydro C_18 _4 μm column (4.6 × 150 mm, Phenomenex, Torrance, CA); running conditions were 0.1% trifluoroacetic acid in 40% methanol as mobile phase, flow of 0.5 mL/min. Detection was performed by photodiode array at 330 nm. The maximum theoretical yield of anthranilate from glucose (^max^Y_Ant/Glc_) was determined by applying elementary mode flux analysis using METATOOL [33].

## Competing interests

The authors declare that they have no competing interests.

## Authors' contributions

VEBH carried out the production experiments and wrote the manuscript. AS, PS and NCV constructed the plasmids and strains. GHC assisted the determination of metabolites. JLBV, AM and FB participated in the design of the study. GG coordinated the study and wrote the manuscript. All authors read and approved the final manuscript.
